# Communicating emotions, but not expressing them privately, reduces moral punishment in a Prisoner’s Dilemma game

**DOI:** 10.1038/s41598-023-41886-9

**Published:** 2023-09-06

**Authors:** Ana Philippsen, Laura Mieth, Axel Buchner, Raoul Bell

**Affiliations:** Department of Experimental Psychology, Heinrich Heine University Düsseldorf, Universitätsstrasse 1, 40225 Düsseldorf, Germany

**Keywords:** Psychology, Human behaviour

## Abstract

The existence of moral punishment, that is, the fact that cooperative people sacrifice resources to punish defecting partners requires an explanation. Potential explanations are that people punish defecting partners to privately express or to communicate their negative emotions in response to the experienced unfairness. If so, then providing participants with alternative ways to privately express or to communicate their emotions should reduce moral punishment. In two experiments, participants interacted with cooperating and defecting partners in a Prisoner’s Dilemma game. After each round, participants communicated their emotions to their partners (Experiments 1 and 2) or only expressed them privately (Experiment 2). Each trial concluded with a costly punishment option. Compared to a no-expression control group, moral punishment was reduced when emotions were communicated to the defecting partner but not when emotions were privately expressed. Moral punishment may thus serve to communicate emotions to defecting partners. However, moral punishment was only reduced but far from being eliminated, suggesting that the communication of emotions does not come close to replacing moral punishment. Furthermore, prompting participants to focus on their emotions had undesirable side-effects: Privately expressing emotions diminished cooperation, enhanced hypocritical punishment (i.e., punishment of defecting partners by defecting participants), and induced an unspecific bias to punish the partners irrespective of their actions.

People have a strong tendency to punish unfair behaviors and are even willing to accept costs to do so^1–7^. As a potential explanation for this puzzling behavior, it has been suggested that moral punishment of unfair behaviors of others is driven by the negative emotional reaction to the perceived unfairness^8–12^. This explanation is supported by evidence showing that the experience of negative emotions can increase punishment rates^13–15^. Conversely, offering players of social dilemma games alternative ways to vent their frustration about an experienced unfairness can reduce punishment rates^16–18^. Moral punishment may thus serve to express or to communicate emotional evaluations of the other’s behavior, hence forth referred to as *emotion communication*. If so, then providing participants with alternative ways to privately express or communicate their emotions should reduce moral punishment. Here, we test these hypotheses by examining how emotion expression and communication affects moral punishment in a Prisoner’s Dilemma game.

Cooperation implies accepting costs for the benefit of others. On average, humans have a rather strong disposition to cooperate with others that prevails even when interacting with strangers in one-shot interactions, albeit the strength of this disposition varies among individuals. An overall strong propensity for cooperation is essential to the evolutionary success of human groups and cultures^19, 20^. Free riders, on the other hand, may exploit the cooperation of others without reciprocating. If too many group members free-ride, cooperation declines and eventually collapses^4, 19, 21^. Hence, a dilemma arises: If each group member only follows their selfish interests, the outcome for the group as a whole is worse than it could have been if every group member had cooperated.

This dilemma between what is in the immediate interest of the individual and what is best for the group is captured in the Prisoner’s Dilemma game^22^. Two players are endowed with a certain amount of money. They can either decide to cooperate by sacrificing part of their endowment for the collective good or they can decide to defect by refraining from cooperation at the other player’s expense. The Prisoner’s Dilemma is defined by its payoff structure (Fig. 1). A defecting player who benefits from a cooperating partner receives the highest payoff. Mutual cooperation leads to a better outcome for both players than mutual defection. A cooperating player who interacts with a defecting partner receives the lowest outcome. At a collective level, cooperation is desirable because mutual cooperation leads to a better outcome for both players than mutual defection. However, at an individual level, it is always more profitable to defect, irrespective of what the other player does. This payoff structure thereby captures the basic dilemma of cooperation. From this clash of individual and collective interests, the free-rider problem arises. Cooperation can only be maintained at a high level in groups and societies if the free rider problem is solved.

**Figure 1:**
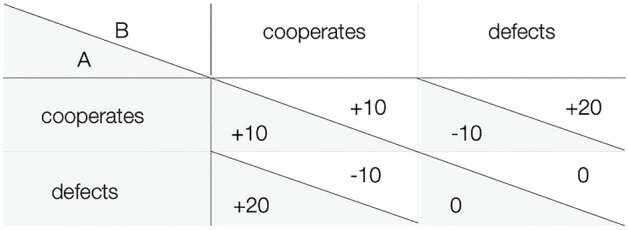
Payoff structure of the Prisoner’s Dilemma game. Values in shaded cells indicate the payoff to Player A, values in white cells indicate the payoff to Player B. The payoffs are displayed as a function of both players’ decisions in the Prisoner’s Dilemma game.

One solution to the free rider problem is moral punishment as it removes the incentive of defection and thereby effectively enforces cooperation^19, 23, 24^. The threat of moral punishment can greatly enhance cooperation rates by reducing free riding^3, 25–32^. However, moral punishment incurs costs to the punisher^4, 33, 34^. Participants have to invest some of their own endowment to deduce points or money from the other player’s account. In repeated interactions, punishers can build a reputation^35–41^ and can thereby directly benefit from their punishment, forcing others to cooperate^42, 43^. However, people use costly punishment even in one-shot interactions in which they cannot build a reputation or benefit in another way from forcing others to cooperate in subsequent rounds^3, 5, 6, 44^. This raises the question: What drives people to punish free riders in one-shot interactions?

As a proximate mechanism, Fehr and Gächter^3^ have proposed that moral punishment is driven by strong negative emotions. Unfair decisions have been found to cause anger in a variety of different social dilemma games^3, 8, 45–47^. People may use costly punishment to express their negative emotional response to the experience of unfairness. Consistent with this view, people who use costly punishment often report anger^8, 14, 18, 31, 48, 49^ and show evidence of emotional arousal in physiological measures^9, 11, 46, 50–52^. Further, Seip et al.^14^ showed that it is not the mere perception of unfairness per se but rather the anger in response to the perception of unfairness that triggers moral punishment. Accordingly, anger has been demonstrated to mediate punishment in several correlation-based analyses^8, 11, 53^. A causal role of anger in moral punishment is supported by findings showing that experimentally induced anger increases punishment rates^13–15, 54^.

In line with the basic tenets of classical catharsis theory^55, 56^, some researchers have suggested that offering participants alternative ways to relieve their anger, referred to as *venting*, may reduce costly punishment^17, 18^. Dickinson and Masclet^18^ demonstrated that different venting methods could significantly reduce subsequent punishment rates in the Public Good game, a multi-player variant of the Prisoner’s Dilemma game. Offering participants the opportunity to rate their experienced emotions (anger, joy and surprise) during a cooling-off waiting phase diminished punishment rates even more than a cooling-off waiting period without emotional ratings but not as much as a high venting condition that included a combination of different venting strategies. These findings suggest that the opportunity to express one’s current emotional state may, to some degree, decrease the need for moral punishment.

In economic games, applying costly punishment is often the only option for players to express their negative emotions. Xiao and Houser^16^ therefore examined how the expression of emotions—as a potential alternative to costly punishment—affected the participants’ behavior in an Ultimatum Game. In the Ultimatum Game, one player, *the proposer,* is endowed with a certain amount of money and can decide to send any proportion of this endowment, ranging from 0 to 100%, to the other player*.* The *responder* can then decide to accept, allowing for the monetary shares to be paid out as proposed, or to reject the offer, causing both players to receive nothing. Rejection in the Ultimatum Game can be interpreted as costly moral punishment as the responder sacrifices money to deny the proposer an unfair share^8, 57, 58^. When players of a one-shot Ultimatum Game were given the option to send written messages to the other player to communicate how they felt, the rejection of unfair offers declined, suggesting that the communication of emotions partly replaced moral punishment. This interpretation, however, is not straightforward because the rejection of offers in the Ultimatum Game could also be interpreted as a decline in cooperation. It thus seems interesting to examine how the communication of emotions affects moral punishment in a paradigm that allows to more clearly distinguish between cooperation and moral punishment.

In the present study, a one-shot simultaneous Prisoner’s Dilemma game was combined with a costly punishment option^59–62^. To assess cooperation and different types of punishment, we used the multinomial cooperation-and-punishment model that has been successfully applied and validated in previous studies^61, 62^. The model serves to distinguish among cooperation, three types of punishment (moral, hypocritical and antisocial punishment) and a punishment bias. Within the model, *moral punishment* is defined as the type of punishment that is exclusively triggered by an unfair interaction in which the participant’s cooperation is exploited by the partner’s defection. This type of punishment can be considered moral because it implies sacrificing resources to enforce norms of cooperation^62^. While our hypotheses mainly pertain to moral punishment, other types of punishment occur in social dilemma games as well^1, 5, 44, 63–71^. Within the model, *hypocritical punishment* is defined as the type of punishment that is exclusively triggered by an interaction in which both partners defect. The purpose of this type of punishment is to enforce a cooperative norm the participants themselves fail to follow^62^. *Antisocial punishment* is defined as the type of punishment that is exclusively triggered by the mismatch between the participant’s defection and the partner’s cooperation. This type of punishment serves to oppose the normative pressure toward cooperation. Finally, people may show an unspecific bias for punishment implying that they punish their partners regardless of the outcome of the Prisoner’s Dilemma game, for instance, when they are distracted from the task^61^, so that a proper measurement model of punishment has to take bias into account.

The aim of the present experiments was to test how the expression and communication of emotions about unfair interactions affects moral punishment. Experiment 1 serves to test whether moral punishment is reduced when participants have an alternative route for communicating their negative emotions about unfair interactions to their partners. To this end, we manipulated between subjects whether participants could communicate their emotional response to the outcome of the Prisoner’s Dilemma game. If moral punishment serves to communicate emotions, moral punishment should be reduced in a condition in which participants can communicate their negative emotions about the outcome of the Prisoner’s Dilemma game to their partners in comparison to a condition in which there is no way of communicating emotions to the partner other than the act of punishment. However, a reduction of moral punishment may also be predicted based on the catharsis account, according to which the mere expression of emotions is already sufficient and communication is not necessary to cause a reduction in punishment. Experiment 2 was designed to test the catharsis account by comparing the effects of an emotion-communication condition to the effects of a private-emotion-expression condition in which emotions were only privately expressed. To anticipate, both experiments demonstrate that moral punishment is reduced—but only by a rather small amount—in the emotion-communication condition in comparison to the control condition. Privately expressing emotions without communicating them had no effect on moral punishment, favoring the emotion-communication account over the catharsis account. No specific hypotheses were derived about the non-moral types of punishment and the punishment bias. Nevertheless, it is interesting to explore whether the effects of emotion expression are closely restricted to moral punishment or whether there are potential harmful side effects of prompting participants to focus on their emotions.

## Experiment 1

### Method

#### Sample

We aimed at collecting at least 200 valid data sets (100 per group) and stopped data collection at the end of the week in which this criterion was reached. The final sample consisted of 203 participants (130 female, 73 male) with a mean age of 22 (*SD* = 4) who were randomly assigned to one of two conditions. One group of participants had the option to communicate their emotions about the Prisoner’s Dilemma game to the partner (*n* = 101) and one group of participants did not have this option (*n* = 102). A sensitivity analysis showed that, with this sample size and 20 punishment decisions per participant, it was possible to detect small effects of *w* = 0.06 with a statistical power of 1 – β = 0.95 at an α level of 0.05 when comparing the moral-punishment parameters between the two conditions^72^. The study was advertised on campus and in social media. Participants received either course credit or a small honorarium as a compensation for participation.

#### Materials and procedure

After consenting to the study, participants were informed that their task was to interact with different partners during a game. At the start of the experiment, they were endowed with 4 € (displayed as 400 cents) which they could invest into the game. Participants were informed that they would play for real money and that they would receive the money in their account at the end of the experiment. Participants played 26 trials of a simultaneous one-shot Prisoner’s Dilemma game with a costly punishment option. The first six trials were training trials. Participants interacted with partners whose responses were determined by a computer program to ensure that half of the partners cooperated and half defected while still presenting trials in random order. The experimental manipulation of the behavior of the partners is a common approach in Experimental Psychology to gain control over confounding factors that may influence an individual’s behavior. The same procedure has been used in many previous studies applying the same task^59–62, 73^ and is similar to the procedures used in other studies on the psychological underpinnings of social cooperation^44, 46, 74, 75^.

**Figure 2:**
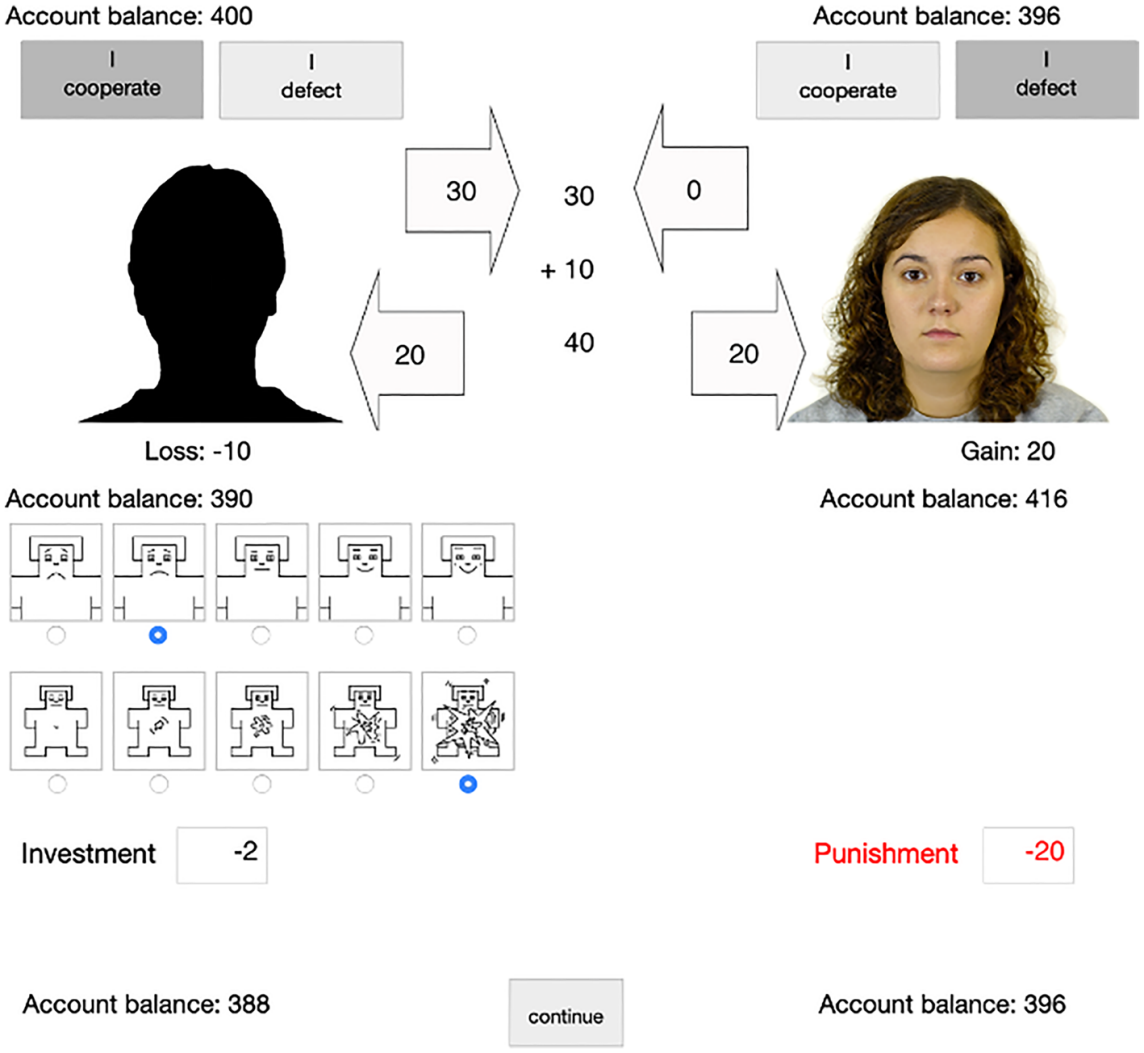
Example trial of the Prisoner’s Dilemma game with costly punishment in Experiment 1. In this example trial, the participant in the emotion-communication condition chose to cooperate while the partner defected, resulting in a loss of 10 cents for the participant (left) and a gain of 20 cents for the partner (right). The participant then used the valence and arousal scales of the Self-Assessment Manikin^77^ to communicate their emotions to the partner. Afterwards, the participant invested two cents to deduce 20 cents from the partner’s account as punishment. The partner’s photograph was randomly selected from the Chicago Face Database^76^.

In each round, participants saw a silhouette on the left side of the screen representing themselves (see Fig. 2). To emphasize the social nature of the game, a color photograph (640 × 480 pixels) of a different partner was displayed on the right side of the screen in each round of the game. The photograph was randomly drawn from a pool of 90 female and 90 male faces of the Chicago Face Database^76^, matching the participants’ gender. These photographs showed the faces of young white adults. All faces had a neutral expression and were shown from a frontal view.

Participants could decide whether they wanted to cooperate or to defect. Cooperating meant to invest 30 cents in a joint business venture while defecting meant to invest nothing. To emphasize the social implications of the behavior in the Prisoner’s Dilemma game (cf.^62^), the two options “I cooperate” and “I defect” were displayed as buttons above both the silhouette and the partner’s photograph. Participants knew that they would make their decision at the same time as their partner and that these decisions affected their payoff.

When the participants had made their decision, the selected option was highlighted. At the same time, the partner’s choice was displayed. The corresponding investments of both partners were displayed in arrows moving from each side to the center of the screen within 750 ms. With a delay of 750 ms, the sum of investments was displayed in-between the two arrows. After another 750 ms, a bonus—corresponding to one third of the sum of investments—was displayed. After 750 ms, the bonus was added to the sum of investments and the resulting total sum in the shared account was revealed. Both partners received half of the money in the shared account irrespective of their investments. After 750 ms, the two shares were represented by two arrows moving away from the center to each side of the screen within 750 ms. Finally, the individual gains or losses as well as the updated account balances were displayed. The game was thus associated with the following outcomes: If both players cooperated by investing 30 cents, a bonus of 20 cents was added to the invested 60 cents so that both the participant and the partner received 40 cents from the total sum of 80 cents, resulting in a gain of 10 cents for each player. If both players defected, none of them gained or lost any money. If one of the players cooperated by investing 30 cents and the other player defected by investing nothing, a bonus of 10 cents was added to the shared account of 30 cents so that both players received 20 cents from the total sum of 40 cents. This resulted in a gain of 20 cents for the defecting player and in a loss of 10 cents for the cooperating player. The payoff structure thus corresponds to that of a typical Prisoner’s Dilemma in which the collective incentive to cooperate clashes with the individual incentive to defect^22^.

After each round of the Prisoner’s Dilemma game, participants were provided with a costly punishment option displayed at the bottom of the screen. Participants were asked to indicate whether they wanted to punish the partner. They could invest either 0 cents if they did not want to punish their partner or 1 to 9 cents from their own account to subtract 10 to 90 cents from their partner’s account. This 1:10 ratio was chosen to facilitate the use of the punishment option. The same ratio has been frequently applied in previous investigations using the multinomial cooperation-and-punishment model^59–62^. One second after participants had confirmed their punishment decision by clicking a “Punishment” button, the updated account balances were shown at the bottom of the screen. Participants could then press a “Continue” button to start the next trial.

At the end of the experiment, participants were thanked, compensated for their participation and debriefed that they had interacted with preprogrammed partners. They were reminded that they could withdraw their consent to the storage and processing of their data without having to accept any detriments but no participant did so. The amount of money paid to the participants varied between 3.50 and 6.60 € (*M* = 5.21, *SD* = 0.78). The experiment took about 12 min on average.

#### Communication of emotions

To examine the effect of emotion communication on punishment, participants were randomly assigned to one of two conditions. One group of participants had no opportunity to express their emotions (no emotion expression). Another group of participants was asked to communicate to their partner how they felt about the interaction in the Prisoner’s Dilemma game before they decided whether to punish the partner (emotion communication). The instructions in the emotion-communication condition explained that participants would be able to communicate their emotions to their partners:

[You] have the opportunity to communicate to your partner how you have felt. Two rating scales are available for this purpose. Please indicate on the first scale how happy or unhappy you have felt. Please indicate on the second scale how relaxed or aroused you have felt. These scales will be displayed to your partner as soon as you confirm your response using the ‘Continue’ button.

The participants rated their emotions on the valence and arousal scales of the *Self-Assessment Manikin*^77^. The two non-verbal scales consist of five pictograms each, one for valence (from 1 = unhappy to 5 = happy) and one for arousal (from 1 = calm to 5 = aroused). Average valence scores were *M* = 2.63 (*SD* = 0.68) in response to defecting partners and *M* = 3.82 (*SD* = 0.64) in response to cooperating partners. Average arousal scores were *M* = 2.36 (*SD* = 0.94) in response to defecting partners and *M* = 2.27 (*SD* = 0.96) in response to cooperating partners.

#### The cooperation-and-punishment model

**Figure 3:**
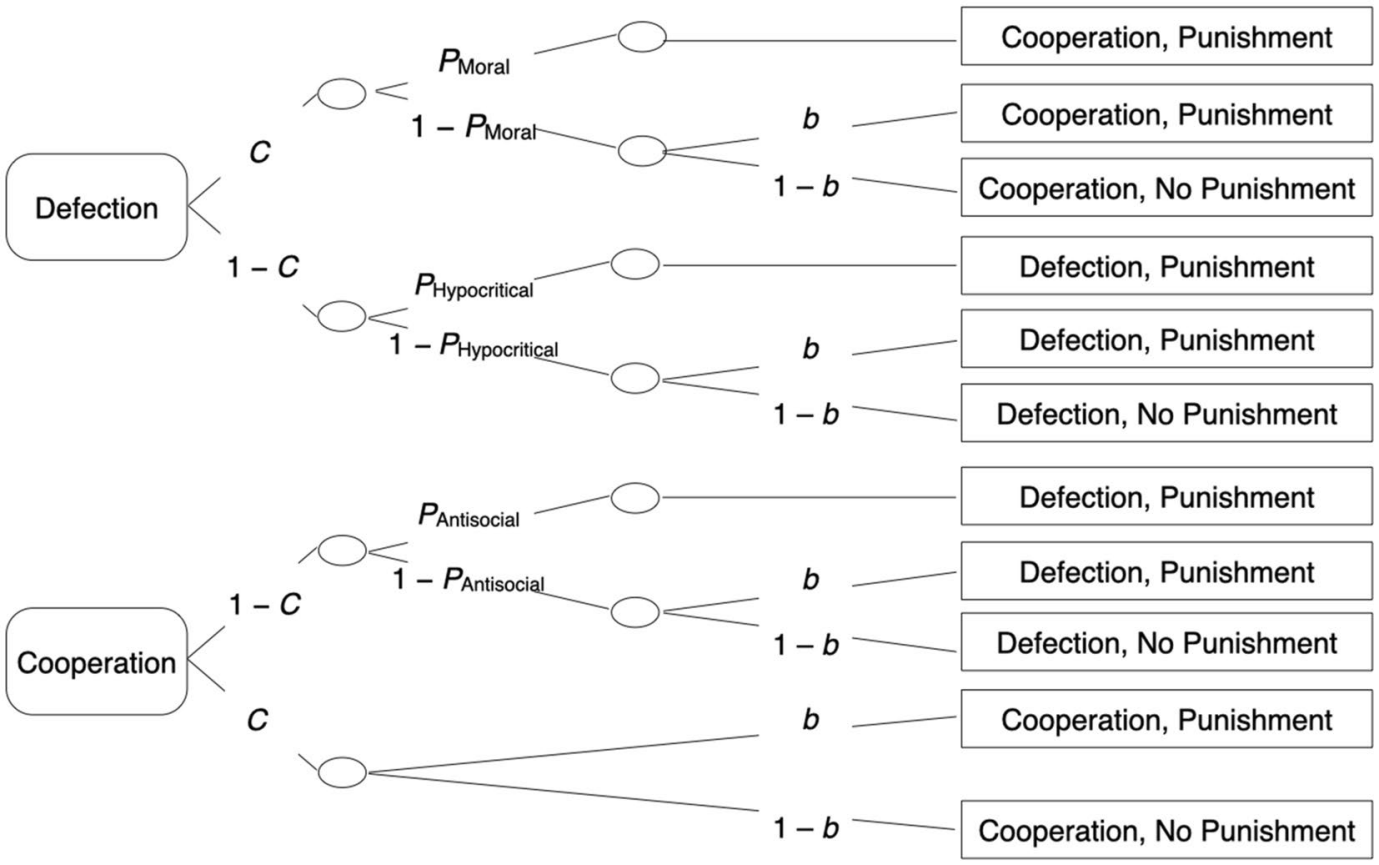
Multinomial cooperation-and-punishment model. Rounded rectangles on the left represent the partner behavior, rectangles on the right represent the participant behavior in a one-shot Prisoner’s Dilemma game with a costly punishment option. Letters along the branches denote the parameters of the model (*C* = cooperative behavior in the Prisoner’s Dilemma game, *P*_Moral_ = moral punishment after unilateral cooperation, *P*_Hypocritical_ = hypocritical punishment after mutual defection, *P*_Antisocial_ = antisocial punishment after unilateral defection; *b* = unspecific punishment bias).

Multinomial models are useful tools to disambiguate observable categorical data by distinguishing among underlying latent processes and their contributions to observed response frequencies in terms of parameter probabilities^78–81^. Computer programs such as *multiTree*^82^ have been developed to test how well a model fits the data, to estimate the model parameters and to test hypotheses directly at the level of the model parameters. Hypotheses tests are performed by introducing parameter restrictions and testing whether the restrictions lead to a significant decrease in how well the model fits the data. The multinomial model used here has already been successfully applied and validated in previous studies^61, 62^. A graphical illustration of the model is shown in Fig. 3. The upper tree in Fig. 3 refers to interactions with defecting partners, the lower tree refers to interactions with cooperating partners.

The first latent process specified in each tree is that participants may choose to cooperate with probability *C* or to defect with the complementary probability 1 – *C*. Given that the participant and the partner decide simultaneously whether to cooperate or to defect, the participant’s cooperation is assumed to be independent of the partner’s cooperation. Therefore, the same parameter *C* is used in both trees of Fig. 3. If the participant’s cooperation is met with the partner’s defection, the participant uses moral punishment with probability *P*_Moral_. When no moral punishment is applied with probability 1 – *P*_Moral_, punishment may still occur due to an unspecific punishment bias with probability *b*. With probability 1 – *b*, no punishment is applied. Following mutual defection, hypocritical punishment may be applied with probability *P*_Hypocritical_. Even if no hypocritical punishment is applied with probability 1 – *P*_Hypocritical_, punishment may still occur due to the punishment bias with probability *b*. With probability 1 – *b*, no punishment is applied. If participant’s defection is met with a partner’s cooperation, antisocial punishment may be applied with probability *P*_Antisocial_. If no antisocial punishment is applied with probability 1 – *P*_Antisocial_, punishment may still occur due to the punishment bias with probability* b*. With probability 1 – *b*, no punishment is applied. Mutual cooperation does not provide any specific reason to punish the partner but punishment may still occur due to the punishment bias *b* (cf.^61, 62^). With probability 1 – *b*, no punishment is applied.

### Results

**Figure 4:**
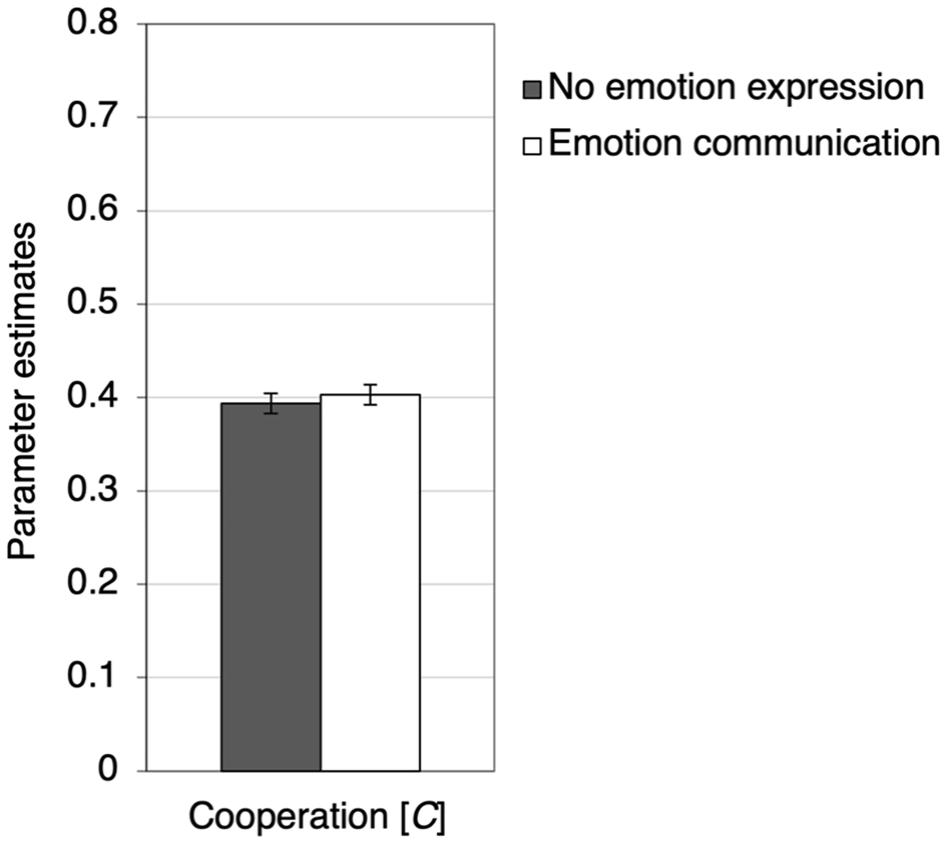
Estimates of the cooperation parameter *C* as a function of whether emotions could be communicated after each round of the Prisoner’s Dilemma game (no emotion expression, emotion communication). The error bars represent standard errors.

The data were analyzed using the multinomial cooperation-and-punishment model (see Fig. 3) to assess how the communication of emotions affects cooperation and punishment. Two instances of the model are needed to analyze the results of Experiment 1, one instance for each condition (no emotion expression, emotion communication). The base model fit the data, *G*^2^(2) = 0.99, *p* = 0.610. The estimates of the cooperation parameter *C* are shown in Fig. 4. Cooperation did not significantly differ between conditions, Δ*G*^2^(1) = 0.37, *p* = 0.543, *w* = 0.01.

**Figure 5:**
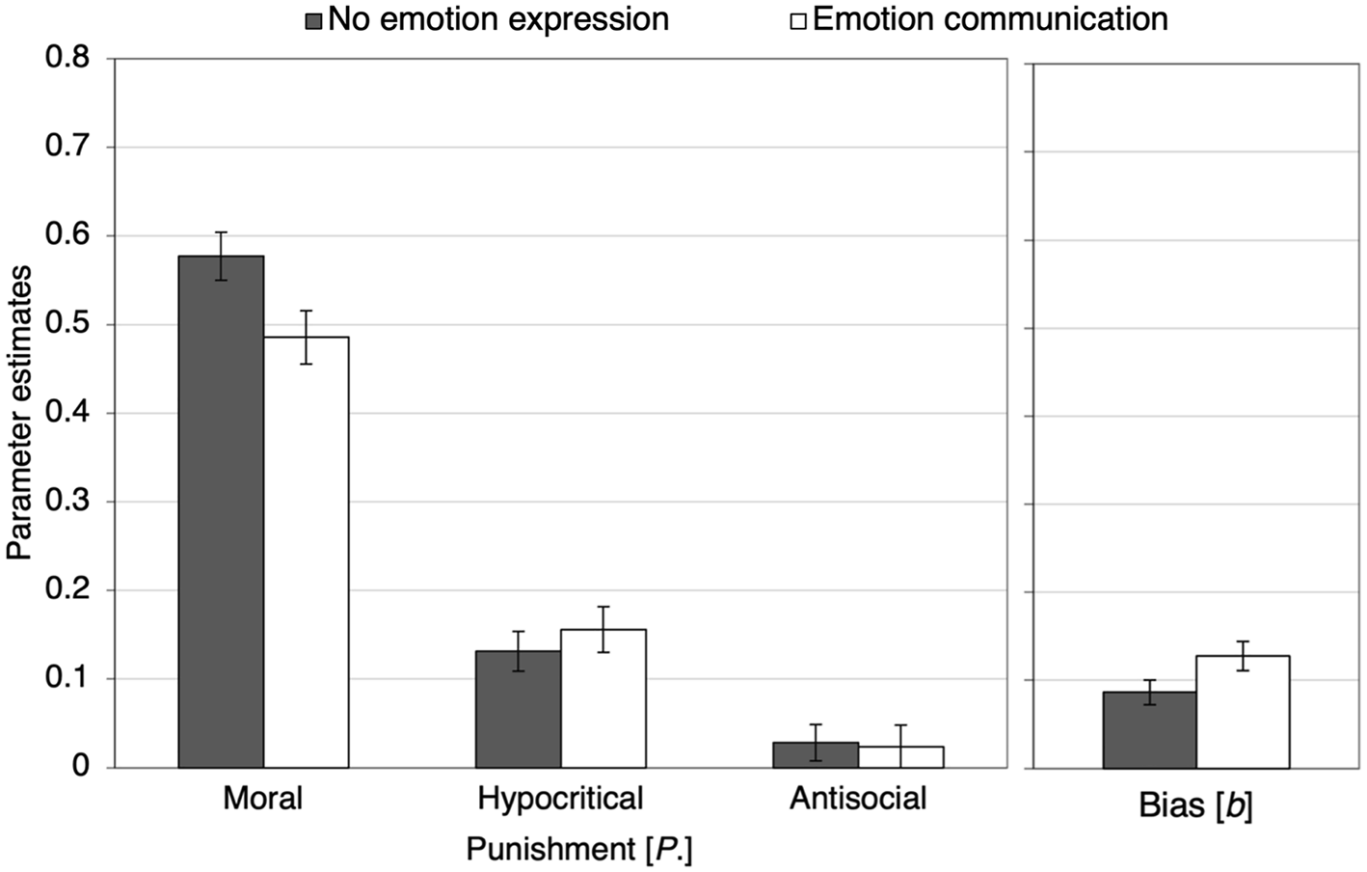
Estimates of the parameters representing moral, hypocritical and antisocial punishment (left panel) and punishment bias (right panel) as a function of whether participants could communicate their emotions about the Prisoner’s Dilemma game before punishing the partners (no emotion expression, emotion communication). The error bars represent standard errors.

Figure 5 displays the results pertaining to costly punishment. The left panel shows the estimates of the parameters representing moral, hypocritical and antisocial punishment. As predicted, moral punishment was significantly reduced when participants had the opportunity to communicate their emotions to their partners in comparison to when they were not given this opportunity, Δ*G*^2^(1) = 5.11, *p* = 0.024, *w* = 0.04. Neither hypocritical, Δ*G*^2^(1) = 0.51, *p* = 0.476, *w* = 0.01, nor antisocial punishment, Δ*G*^2^(1) = 0.02, *p* = 0.881, *w* < 0.01, differed significantly between conditions. The right panel shows the estimates of the punishment-bias parameter. At a descriptive level, the punishment bias was enhanced in the emotion-communication condition in comparison to the no-emotion-expression condition but this difference was not significant, Δ*G*^2^(1) = 3.64, *p* = 0.056, *w* = 0.03.

### Discussion

The aim of Experiment 1 was to test the prediction of the emotion-communication account of moral punishment according to which moral punishment should be reduced when participants have an alternative route of communicating their emotions to their partners after each round of the Prisoner’s Dilemma game. In line with this prediction, the communication of emotions significantly reduced moral punishment relative to a condition in which participants had no opportunity to express their emotions other than punishment. This finding suggests that one function of moral punishment is the communication of negative emotions in response to the partner’s unilateral defection. However, moral punishment was only reduced, but far from being eliminated in the emotion-communication condition. The communication of emotions thus reduces the need for moral punishment but does not come close to replacing it. The effect of emotion communication was specific to moral punishment. Hypocritical punishment of defecting partners was not affected by communicating one’s emotions prior to punishment decisions. Similarly, the communication of emotions did not affect antisocial punishment of cooperating partners. Within the limits of the sensitivity of the statistical tests used in Experiment 1, these findings suggest that the communication of emotions does not seem to be a relevant factor in hypocritical and antisocial punishment.

## Experiment 2

Given that the effect of communicating emotions on moral punishment was relatively small in Experiment 1, an important aim of Experiment 2 was to test whether this effect can be replicated. The most important aim of Experiment 2, however, was to test whether this effect, if replicated, was due to the communication of negative emotions to the partner, as implied by the emotion-communication account, or whether it was simply due to the mere expression of negative emotions, as implied by the catharsis account. Given that, in Experiment 1, an emotion-communication condition was contrasted with a control condition in which one’s emotions could not be expressed, it is impossible to distinguish between these two accounts. The results of Experiment 1 are thus not only consistent with the emotion-communication account; the results are equally compatible with the catharsis account according to which the mere expression of emotions suffices to reduce the need for moral punishment.

Both accounts have received some support in previous studies. For instance, Xiao and Houser^16^ found that offering responders an opportunity to communicate their emotions to proposers in an Ultimatum Game diminished rejection rates. This finding supports the assumption that one function of moral punishment is to communicate to the proposers that their behavior was inadequate under a cooperative norm. According to this account, the component of moral punishment in question is directed at regulating *others’* behaviors. By contrast, Dickinson and Masclet^18^ found a significant effect on punishment rates in a Public Goods game when participants expressed their emotions privately in written messages they knew would never be sent, supporting the assumption that a private self-centered process underlies the application of costly punishment with the intention to regulate one’s *own* emotions. Experiment 2 was designed to distinguish between these contrasting accounts. To this end, the emotion-communication condition was compared to a no-emotion-expression and to a private-emotion-expression condition. If the effects of emotional responses on moral punishment are due to a self-centered venting process, moral punishment should be reduced in both the emotion-communication condition and the private-emotion-expression condition in comparison to the control condition without emotion expression. If it is the communication of emotions that underlies the effect, then moral punishment should be reduced only in the emotion-communication condition but not in the private-emotion-expression condition in comparison to the no-emotion-expression control condition. Experiment 2 also addresses another limitation of Experiment 1, namely that punishment was unilateral. Participants could punish their partners but did not receive any punishment from the partners. Previous studies^59–62^ have shown that antisocial punishment rates are quite low under these conditions suggesting that the experience of being punished increases antisocial punishment which is in line with the idea that antisocial punishment serves to oppose the normative pressure toward cooperation. Consistent with these previous findings, antisocial punishment occurred only with a comparatively low probability in the present Experiment 1. It thus seemed interesting to explore whether the expression of emotions has an effect on antisocial punishment when antisocial punishment occurs with a higher probability which represents more favorable conditions for finding an effect if it exists. Also, we deliberately increased the sample size and, thus, the sensitivity of the statistical tests, in Experiment 2 relative to the sample size of Experiment 1 such that it was possible to detect even small effects on antisocial punishment, hypocritical punishment and the punishment bias. This seemed important given that, at a descriptive level, hypocritical punishment and the punishment bias were increased in the emotion-communication condition compared to the no-emotion-expression condition in Experiment 1. As a more ecologically valid form of communicating emotions, we asked participants to communicate their emotions in the way they were used to from everyday life, that is, by using the emojis in the design determined by their computer’s operating system rather than the Self-Assessment Manikin that we had used in Experiment 1.

### Method

#### Sample

Given that the aim of Experiment 2 was to further dissect the effect of emotion-expression on moral punishment, we presumed the population effect size of interest to be half the size of the sample effect observed in Experiment 1 (*w* = 0.04). An a-priori power analysis in G*Power^72^ showed that with an α level of 0.05 and 20 punishment decisions in the Prisoner’s Dilemma game, a sample size of *n* = 1625 was necessary to detect an effect of emotion-expression on moral punishment of *w* = 0.02 with a statistical power of 1 – β = 0.95. To achieve such a large sample size, the experiment was performed online. Participants were recruited via the online research panel provider *mingle.* Of those participants who had started the Prisoner’s Dilemma game, 199 withdrew from the experiment, 44 data files were incomplete and 121 data files had to be removed due to double participation. The final sample consisted of 1681 participants (720 female, 957 male and 4 diverse), aged 18 to 87 years (*M* = 53, *SD* = 16).

#### Materials and procedure

**Figure 6:**
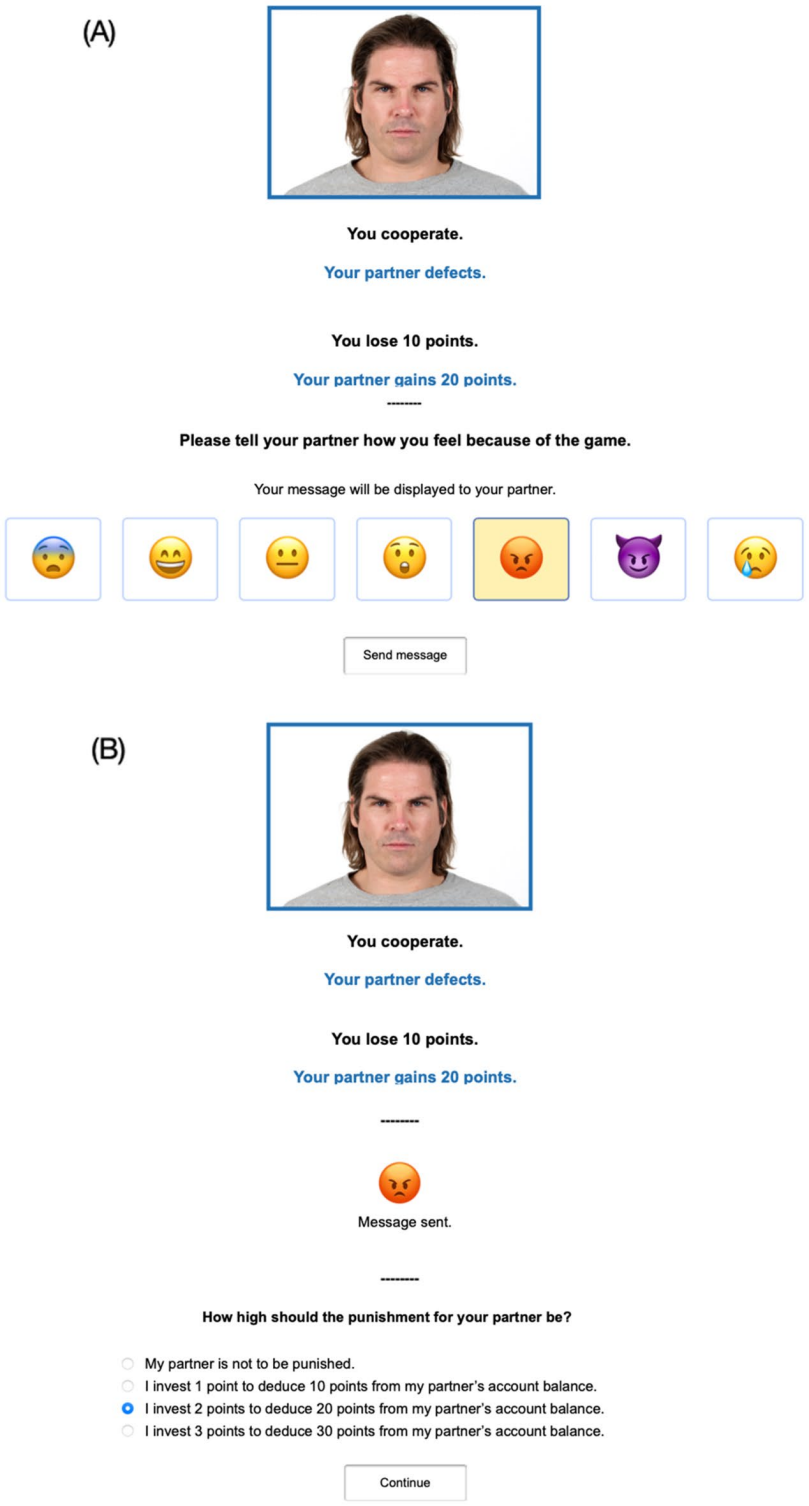
Example trial of the Prisoner’s Dilemma game with costly punishment in the emotion-communication condition in Experiment 2. The cooperating participant chose to send the angry emoji to the defecting partner (**A**) and then decided to invest two points to deduce 20 points from the partner’s account (**B**). The partner’s photograph was randomly selected from the Chicago Face Database^76^.

Switching from the laboratory setting of Experiment 1 to an online format required a few adjustments to the procedure. Participants of the online panel provider *mingle* are used to being compensated with points that can be exchanged for vouchers, charity donations or bank transfers. Therefore, participants were informed that they were playing for points (1 point = 0.01 € or 1 cent) that they would receive from *mingle* after the study was completed. At the start of the game, participants were endowed with 100 points (corresponding to 1 € or 100 cents). As in Experiment 1, participants played 26 rounds of the one-shot simultaneous Prisoner’s Dilemma game (Fig. 6). The first six trials were training trials. Photographs of 26 white adult faces were selected from the Chicago Face Database^76^. Half of the faces were female. The partner’s behavior (cooperation, defection) was again randomly determined. The payoff structure was the same as in Experiment 1.

The procedure was similar to the procedure of Experiment 1 but was adjusted to the online environment to ensure that the information would be displayed smoothly on the participants’ personal computers. Each trial started with the presentation of the participant’s current account balance in the middle of the browser window. After having clicked on a “Continue” button, participants saw the partner’s photograph (266 × 186 pixels) in the middle of the screen. The photograph had a blue frame (4 pixels). The photograph remained visible on screen until the end of the trial. Participants selected whether to cooperate or to defect. Upon clicking a “Continue” button, the participants received written feedback about their own decision (e.g., “You cooperate.”) and their partner’s decision (e.g., “Your partner defects.”) and were informed about the monetary consequences of these decisions for both players (e.g., “You lose 10 points.” And “Your partner gains 20 points.”). Statements referring to the participant were shown in black while statements referring to the partner were shown in blue, corresponding to the blue frame around the partner’s photograph.

The feedback about the outcomes of the Prisoner’s Dilemma game remained visible until the end of the trial. Upon clicking a “Continue” button, participants were asked to make a punishment decision. To simplify the procedure for the online environment, a maximum of up to three points could be invested to subtract between 10 and 30 points from the partner’s account. Other than in Experiment 1, punishment was not unilateral. Participants were informed that the partners had the same punishment option as the participants. The participant’s and the partner’s punishment decisions were displayed simultaneously. To approximate the typical behavior of real players, the partners were programmed to punish the unilateral defection of the participants by investing a random amount between 1 and 3 points to deduce between 10 and 30 points from the participant’s account. Participants received immediate feedback about their own punishment decision (e.g., “You invest 2 points to punish your partner.”) and its effect on the partner’s account (e.g., “20 points are deducted from your partner’s account.”) as well as about the partner’s punishment decision (e.g., “Your partner does not punish you.”) and its effect on the participant’s account (e.g., “No fine will be deducted from your account.”). Again, statements referring to the participant were shown in black while statements referring to the partner were shown in blue.

Participants could then start the next trial by clicking a *“*Continue*”* button. On average, participants acquired a final account balance of 78 (*SD* = 32) points. The experiment took about 18 min.

#### Emotion expression and communication

Participants were randomly assigned to one of three groups: Participants in the *private-emotion-expression* condition (*n* = 576) privately expressed their emotional state without communicating with their partner. Participants in the *emotion*-*communication* condition (*n* = 541) sent a message about their emotional state to their partners. Participants in the control condition (*n* = 564) had no opportunity to express or to communicate their emotions. In the private-emotion-expression condition, participants were asked to express how they felt after each Prisoner’s Dilemma interaction. They were reassured that this information would not be shared with their partners. Participants selected one of seven emojis, ordered in the same random horizontal array in each trial, and submitted their answer using a button labeled “save emotional state”. After having confirmed their response, the selected emoji was displayed along with a statement confirming that the emotional state had been saved. In the emotion-communication condition, participants were instructed to communicate to their partner how they felt after the interaction. As in the private-emotion-expression condition, participants selected one of the seven emojis, ordered in the same random horizontal array in each trial, and submitted their answer using a button labeled “send message”. The subsequent feedback confirmed that the message had been sent.

In order to express or to communicate their emotions, participants could choose among seven emojis expressing *anger, sadness, joy, surprise, fear, schadenfreude* and a *neutral* state. The emojis were selected based on a norming study (*N* = 16) in which participants were asked to choose, from a selection of suitable emojis, the ones that best expressed these emotions. The emojis that were displayed were those offered by the participant’s individual operating system which allowed participants to use the same emojis as in their everyday online communication. Participants were instructed either to privately express their emotions or to communicate their emotions to their partner but they were not instructed about the particular type of emotion they were supposed to express. Instead, they could use the available emojis in any way they wanted in order to express or communicate their emotions. The most frequently selected emoji after an interaction with a defecting partner was the one expressing a neutral state (25%), closely followed by the angry emoji (20%). After having interacted with a cooperating partner, the majority of the participants (52%) selected the happy emoji. The selected emoji remained visible during the punishment decision until the end of the trial.

### Ethical approval and consent to participate

Both experiments reported here were conducted in accordance with the guidelines laid down in the Declaration of Helsinki and by the German Research Foundation (DFG) including confidentiality of data and personal conduct. Informed consent was obtained prior to participation. For the noninvasive, purely behavioral research reported in the present series of experiments which carried no risk for the participants, a formal approval by the institution’s ethical board is not legally required in Germany (see: https://www.dfg.de/en/research_funding/faq/faq_humanities_social_science/index.html).

### Results

**Figure 7:**
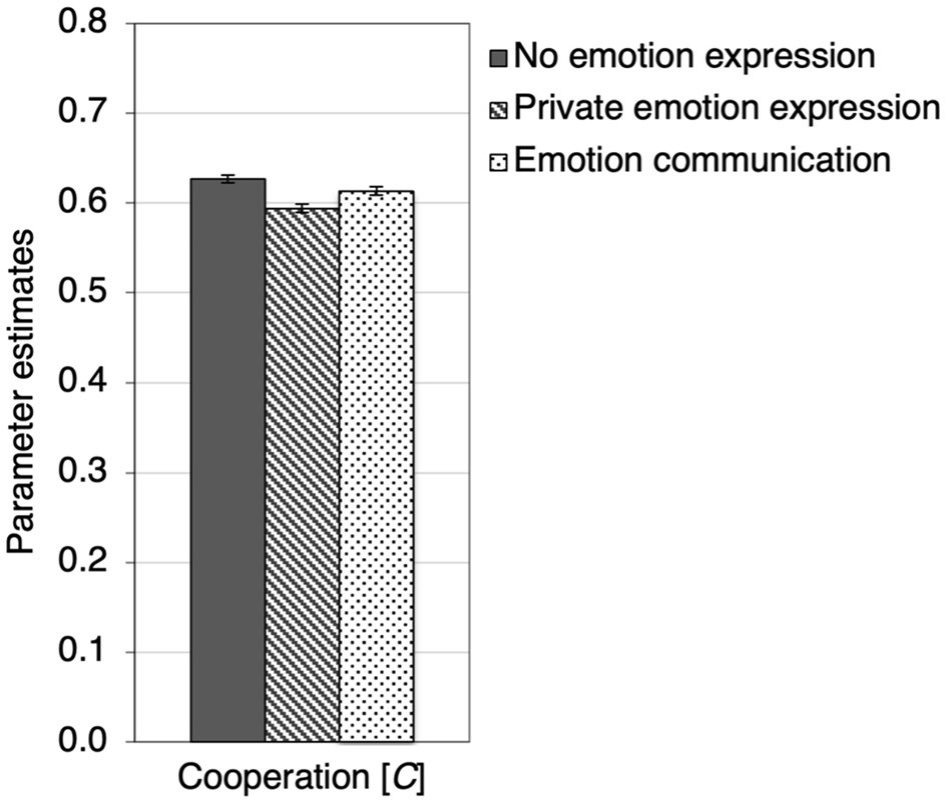
Estimates of the cooperation parameter *C* which specifies the probability of cooperation as a function of whether emotions were expressed or communicated after each round of the Prisoner’s Dilemma game (no emotion expression, private emotion expression, emotion communication). Error bars represent standard errors.

As in Experiment 1, we used the cooperation-and-punishment model illustrated in Fig. 3 to disentangle cooperation, the different types of punishment and the punishment bias. To analyze the results of Experiment 2 we needed three instances of the model illustrated in Fig. 3, one instance for each condition (no emotion expression, private emotion expression, emotion communication). The base model fit the data, *G*^2^(3) = 2.58, *p* = 0.460. The estimates of the cooperation parameter *C* are depicted in Fig. 7. In both the private-emotion-expression condition, Δ*G*^2^(1) = 25.73, *p* < 0.001, *w* = 0.03, and the emotion-communication condition, Δ*G*^2^(1) = 4.37, *p* = 0.037, *w* = 0.01, cooperation decreased in comparison to the no-emotion-expression control condition, suggesting that requiring participants to focus on their emotions about the Prisoner’s Dilemma game had detrimental effects on cooperation. Private emotion expression reduced cooperation even further than emotion communication, Δ*G*^2^(1) = 8.52, *p* = 0.004, *w* = 0.02.

**Figure 8:**
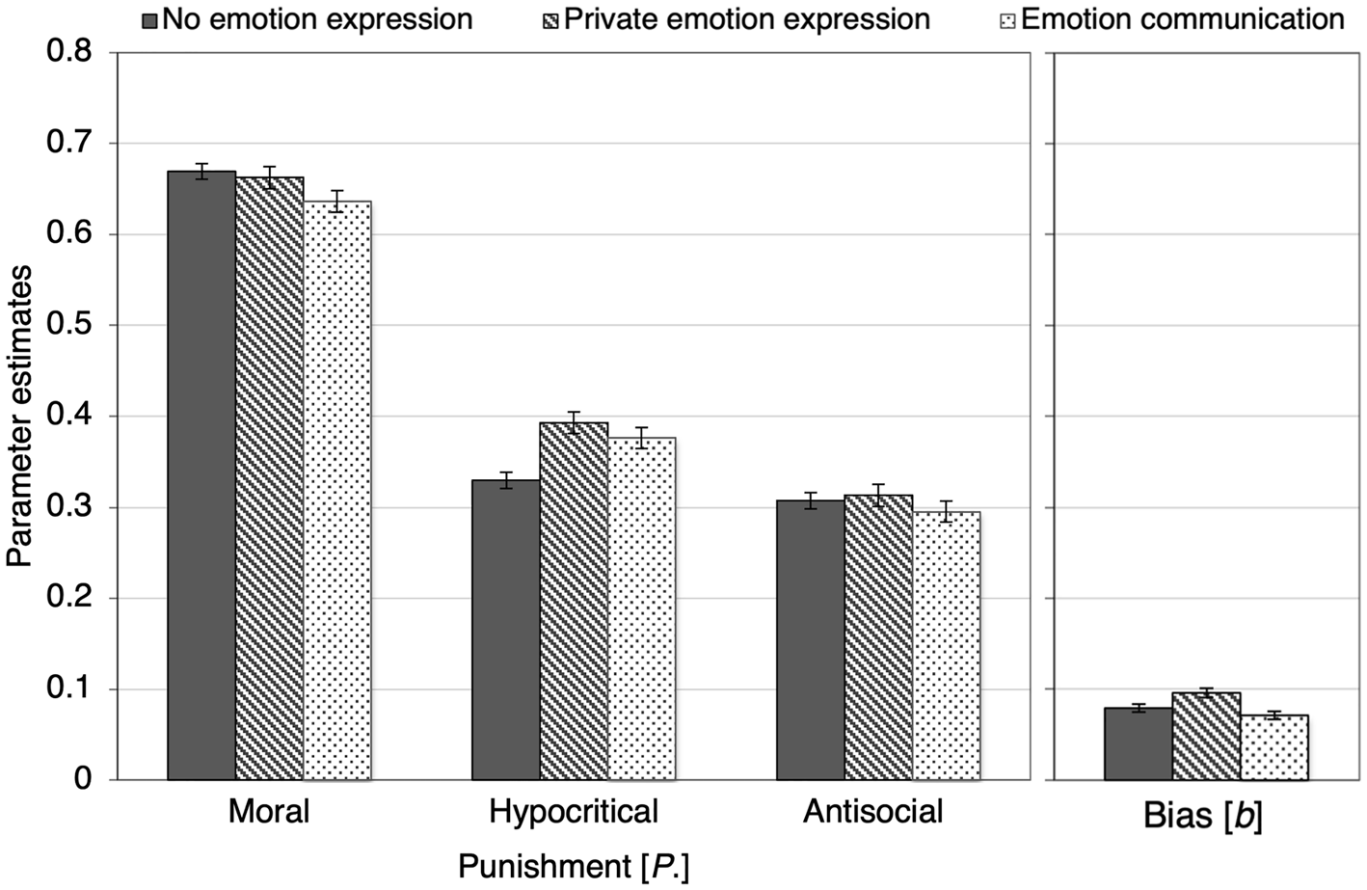
Estimates of parameters representing moral, hypocritical and antisocial punishment (left panel) and punishment bias (right panel) as a function of whether participants could express or communicate their emotions about the Prisoner’s Dilemma game before punishing the partners (no emotion expression, private emotion expression, emotion communication). The error bars represent standard errors.

Figure 8 shows the results pertaining to costly punishment. In line with the main finding of Experiment 1, moral punishment was significantly decreased when participants had the opportunity to communicate their emotions to their partners in comparison to both the no-emotion-expression control condition, Δ*G*^2^(1) = 7.19, *p* = 0.007, *w* = 0.01 and the private-emotion-expression condition, Δ*G*^2^ (1) = 4.32, *p* = 0.038, *w* = 0.01. By contrast, privately expressing emotions did not reduce moral punishment relative to the no-emotion-expression control condition, Δ*G*^2^(1) = 0.32, *p* = 0.573, *w* < 0.01. Hypocritical punishment was enhanced in both the private-emotion-expression condition, Δ*G*^2^(1) = 14.01, *p* < 0.001, *w* = 0.02, and the emotion-communication condition, Δ*G*^2^(1) = 7.55, *p* = 0.006, *w* = 0.01, relative to the no-emotion-expression control condition, while there was no difference between the private-emotion expression condition and the emotion-communication condition, Δ*G*^2^(1) = 1.00, *p* = 0.318, *w* = 0.01.

In line with the results of Experiment 1, there was no effect of emotion expression or communication on antisocial punishment, Δ*G*^2^(2) = 1.22, *p* = 0.543, *w* = 0.01. Communicating one’s emotions did not significantly affect the punishment bias compared to the no-emotion-expression control condition, Δ*G*^2^(1) = 1.60, *p* = 0.206, *w* = 0.01. However, expressing one’s emotions privately significantly increased the punishment bias compared to the no-emotion-expression control condition, Δ*G*^2^(1) = 6.23, *p* = 0.013, *w* = 0.01, and the emotion-communication condition, Δ*G*^2^(1) = 13.81, *p* < 0.001, *w* = 0.02.

### Discussion

The main result of Experiment 1 was replicated in Experiment 2. Communicating one’s emotions to the partner prior to the punishment decision attenuated moral punishment. Extending the results of Experiment 1, Experiment 2 demonstrates that moral punishment is attenuated only when the emotions can be *communicated* to the interaction partner. The fact that moral punishment was not attenuated when participants expressed their emotions privately indicates that the emotional function of moral punishment is other- rather than self-directed.

The pattern of findings is thus consistent with the emotion-communication account according to which moral punishment serves to communicate one’s emotional state in response to the outcome of the Prisoner’s Dilemma game to the partner. The fact that privately expressing emotions had no effect on moral punishment provides evidence against the catharsis account. It seems important to note that moral punishment was only reduced and far from being abolished in the emotion-communication condition. This aspect of the present results suggests that there are other functions of moral punishment in addition to communicating one’s emotions—such as that of reducing the unfair payoff imbalance (cf.^83, 84^)—that are very likely much more important than the communication of emotions.

The overall level of cooperation was considerably higher in Experiment 2 than in Experiment 1. The most likely explanation of this difference is that participants could punish their partners but did not receive any punishment from the partners in Experiment 1. In Experiment 2, by contrast, the partners punished the participants for unilateral defection. It is thus reasonable to assume that the moral punishment of the participants’ unilateral defection enforced more cooperation from participants in Experiment 2, consistent with the role of moral punishment in enforcing cooperation^59, 60, 62^.

By increasing the sample size, we were better able to detect even smaller effects of the communication or expression of emotions in Experiment 2 compared to Experiment 1. This increased sensitivity of the statistical tests may be the reason why some of the effects that were absent or only present at a descriptive level in Experiment 1 reached statistical significance in Experiment 2. First of all, both, expressing one’s emotions privately and communicating one’s emotions, reduced cooperation in the Prisoner’s Dilemma game relative to the no-emotion-expression control condition. Secondly, there was an increase in hypocritical punishment in both the private-emotion-expression condition and the emotion-communication condition relative to the no-emotion-expression control condition. Thirdly, privately expressing one’s emotions also increased the punishment bias relative to the no-emotion-expression control condition, suggesting that privately focusing on one’s emotions may increase the likelihood of punishment regardless of the outcome of the preceding interaction. These findings highlight that prompting participants to focus on emotions and thereby increasing the emotional saliency of the outcomes of the Prisoner’s Dilemma game does not only have desirable effects on social interactions.

## General discussion

People accept costs to punish uncooperative individuals even in one-shot interactions in which punishment cannot yield any direct personal benefits (e.g.,^5, 65^). The question is why people show this economically irrational behavior. Fehr and Gächter^3^ have proposed that this moral form of punishment is driven by a negative emotional response to perceived unfairness. Therefore, negative emotions are assumed to represent a key driving force behind moral punishment. Accordingly, a bulk of studies has linked the application of punishment to self-reports (e.g.,^8, 14^) or physiological indices^50, 52^ of anger. The main aim of the present study was to test whether the expression or communication of emotions may reduce moral punishment. Both experiments confirm that the communication of emotions reduces moral punishment.

Two accounts were distinguished. According to the *emotion-communication account*, moral punishment serves to communicate one’s discontent with a social interaction to the interaction partner. According to the emotion-communication account, providing participants with an alternative way of communicating their emotions should reduce moral punishment. By contrast, privately expressing emotions should be ineffective in reducing moral punishment. According to the *catharsis account*, a reduction of moral punishment relative to the control condition should already be observed when participants privately vent their emotions. Therefore, the catharsis account implies that moral punishment should be reduced in both the emotion-communication condition and the private-emotion-expression condition relative to the no-emotion-expression condition. In line with the emotion-communication account, moral punishment was attenuated when participants were provided with an option to *communicate* their emotions about the Prisoner’s Dilemma game to their partners in Experiments 1 and 2. By contrast, privately expressing one’s emotions did not significantly attenuate moral punishment in Experiment 2 despite the large sample size and, thus, the considerable sensitivity of the relevant statistical test. The pattern of results thus provides evidence against the catharsis account according to which punishment serves to regulate one’s own emotions by venting frustration (cf.^18^) and in favor of the assumption that punishment is instrumental in regulating the other’s behavior, possibly by signaling a negative emotional evaluation of unilateral defection with the purpose to enforce cooperation even if one cannot directly benefit from it^16, 85^.

It is also important to note that both experiments reported here consistently show that the effect of the communication of emotions on moral punishment is only small. A high level of moral punishment remained even in the emotion-communication condition. This indicates that an opportunity to communicate emotions does not come close to replacing moral punishment. However, while emotion communication appears to play only a minor role for moral punishment, this does not entail that emotions per se play a minor role. As previous studies clearly demonstrate, emotions, particularly anger, constitute a strong motivator of costly moral punishment (e.g.,^11, 14, 52^). However, the mere communication of emotions, in contrast to moral punishment, is not effective in removing the unfair payoff differences resulting from the Prisoner’s Dilemma game and might therefore not represent a valid alternative for this emotionally driven form of punishment.

An interesting side finding is that paying attention to emotions, particularly expressing emotions privately without being able to communicate them, had a range of undesirable effects on cooperation and punishment in Experiment 2. Focusing on one’s emotions about the outcomes of the Prisoner’s Dilemma game—especially when they were only privately expressed—had a negative impact on one’s willingness to engage in cooperation, possibly because the focus on emotions emphasized the fact that the payoff structure of the Prisoner’s Dilemma game is constructed in such a way that the individual outcome of the game is always more negative after cooperation (see Fig. 1). Furthermore, expressing and communicating emotions increased hypocritical punishment and privately expressing emotions induced a punishment bias. These findings indicate that integrating emotional responses into the game paradigm may sometimes amplify rather than defuse negative social interactions. These findings go against the predictions of catharsis theory^55, 56^ according to which an opportunity to vent one’s emotional frustration by expressing it should have had attenuating effects on punishment (cf.^18, 86^). This suggests that prompting participants to focus on their emotions—which might otherwise have been less salient—may have social costs. It is a fascinating avenue for future research to further explore the possibility that making emotions salient may have detrimental effects on social interactions.

In line with the research methods of Experimental Psychology^44, 46, 59–62, 73, 74^, the focus of the present study lies on the individual’s cognition and behavior. Therefore, the behavior of the partner is seen as an extraneous influence that is experimentally controlled by factoring it in the design. Furthermore, in the present experiments, participants were not only informed about the raw incentive structure of the game but were also presented with a situation that was rich in social cues (including, for example, the partners’ faces). This is different from Experimental Economics in which the focus is often on how the incentive structure affects the interactions of dyads or groups of individuals interacting with each other. The fact that the participants cooperate with their partners and punish their partners even when cooperation and punishment go against their financial interests, as well as the fact that the communication of emotions to the partners but not the private expression of emotions affected moral punishment, suggests that the present paradigm taps into mechanisms of social interactions. This is in line with recent experimental findings demonstrating that beliefs about the human versus preprogrammed nature of partners has surprisingly little effects on the behavior in economic games^87, 88^. Nevertheless, it is of course an important goal for future research to explore whether the present conclusions hold in more ecologically realistic settings.

## Conclusion

To summarize, two experiments were performed to test the effects of the expression and communication of emotions on moral punishment. In line with the emotion-communication account, moral punishment was attenuated when participants were provided with an alternative way of communicating their negative emotions in response to the outcomes of the Prisoner’s Dilemma game. The effects on moral punishment were only present when the emotions were communicated to the partner and absent when the emotions were merely privately expressed, emphasizing the communicative role of moral punishment. Still, moral punishment was attenuated but did not come close to being abolished by the communication of emotions, suggesting that communicating emotions is only a minor component of moral punishment.

## Data Availability

We provide the data used in our analyses via the Open Science Framework. The data are publicly available at https://osf.io/z8fwh/.
